# Correlation of Trochanter-Shaft Angle in Selection of Entry Site in Antegrade Intramedullary Femoral Nail

**DOI:** 10.5402/2012/431374

**Published:** 2012-05-17

**Authors:** O. P. Lakhwani

**Affiliations:** Department of Orthopedics Surgery, ESIC-Postgraduate Institute of Medical Sciences and Research, New Delhi 110015, India

## Abstract

*Background.* Selection of entry point for nail insertion is controversial and lack firm anatomical basis. The study is done to analyze the proximal anthropometry of femur and measure the Trochanter-Shaft Angle to find its relation and significance in selection of entry point for antegrade uniplanar femoral nail. *Materials and Methods.* Study involves the measurement of trochanter-shaft angle and other anthropometric measurements on 50 dry femora and on digital radiogram. *Results.* Trochanter-Shaft angle ranges between 5–17 degrees in anthropometric study and 4–14 degrees in radiological study. Over all in 27 cases (54%), exit points of reamur fall in the middle quadrant in sagittal and coronal plane, which corresponds to the T-S angle of 6–12 degrees. *Discussion and Conclusion.* Proximal femoral Anthropometry and Trochanter-shaft angle is variable; hence it is difficult to fix any anatomical point as a universal entry point for antegrade femoral nail insertion. Trochanter shaft angle (TSA) can be well accessed radiologically and serve as a guide for selection of proper entry point.for safe nail insertion. *Clinical Relevance.* Individual variations in the proximal femur anatomy for safe nail insertion can be correlated with Trochanter shaft angle to serve safe entry site.

## 1. Introduction

Selection of entry point for antegrade femoral interlocking nail is controversial, and there still is debate as to the most appropriate starting point [1–4]. Piriformis fossa [5–7] has been advocated as traditional entry point. However, complications [7–9] have been reported with traditional Piriformis fossa as a starting point. This has led to interest in the off-axis entry point, but to some extent, this off-axis entry used is not clear as this results in high femoral strain during insertion.

Moreover proximal femoral anthropometry and anatomy of greater trochanter has different dimension [4, 10, 11]; hence it is difficult to define the universal entry point piriformis fossa, greater trochanter, or any other site for antegrade intramedullary nailing. The least starining and anatomical point for safe nail insertion should be in the line of medullary canal of femur, which can be determined proximally on trochanteric area by measuring Trochanter-shaft angle (TSA). Therefore, the present study is undertaken to measure the trochanter shaft angle and develop the anatomical and radiological basis for it.

## 2. Materials and Methods

The Study involves the 50 dry adult cadaveric femora in anatomy museum and biomechanical laboratory. Adult femoral bones that were nonfractured having no bony abnormalities were selected in study. Among 50 femora, 24 were of Right side and 26 Left sides. On these selected femora, anthropometric and radiological measurements were taken with the help of anthropometric instruments—Anthropometric Board, Verniers caliper, adding caliper, Goniometer, Marker pen, and Digital Radiography setup. Femoral medullary canal Reamer, standard uniplaner bent nail was used with the curvature in the anteroposterior plane to follow curvature of medullary canal.

**Figure 1:**
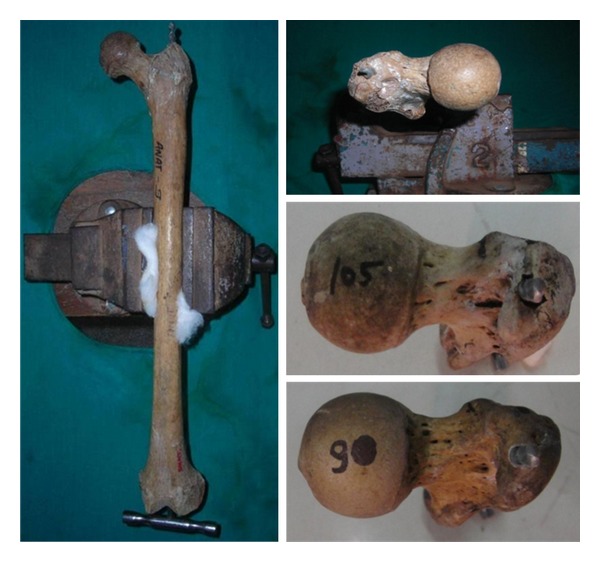
Showing femur mounted over frame with remur passed through intercondylar area in medullary canal and Superior view of proximal femur with various exit points.

The study comprises two sets of methodology—anthropometric measurements of Trochanter shaft angle on adult femora and on Computerized digital radiogram of the dry cadaveric femora. measurements were taken after nullifying the angle of torsion and in reference to antegrade femoral intramedullary nail insertion. Selected dry femur bones were fixed on Table 1 with help of holding device. Retrograde reaming was inserted through midpoint of intercondylar notch as a starting point and advanced to exit through the proximal-superior end of femur (Figure 1). This exit point was noted in coronal and sagittal plane on proximal and superior aspect of femur by dividing the proximal superior femur (trochanter and adjacent area) in 9 quadrants area.

**Figure 2:**
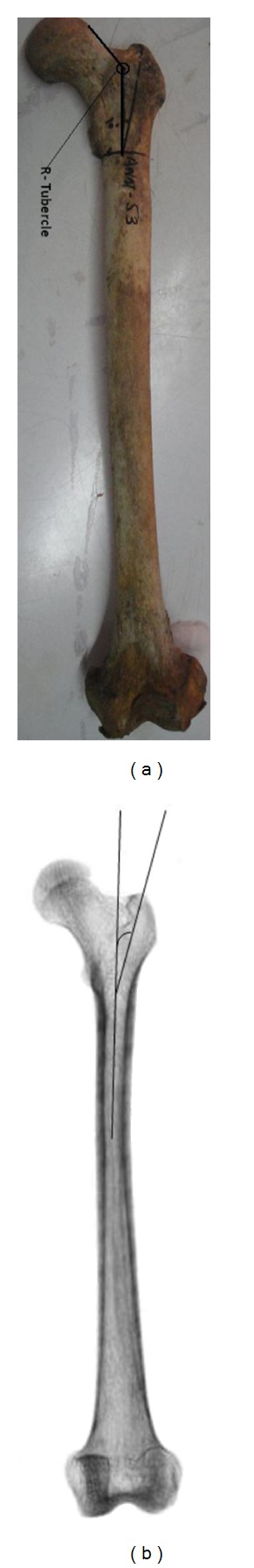
Anthropometric and radiological measurement of trochanter-shaft angle.

Trochanter-Shaft Angle (TSA) is measured by drawing a vertical line along the midline of the shaft. The highest point on the Greater trochanter was selected that can be seen on dry bones and digital radiogram easily; second line is drawn joining this point to the mid-line of the shaft at the level of base of lesser trochanter (which is a transitional zone of cortical and cancellous bone and junction of trochanter to shaft). The angle between the two lines is measured in degrees as Trochanter-shaft angle and named as “R”-angle. Measurement of Trochanter-shaft angle of anthropometric study was compared and analysed with the data taken-up by the digital radiograms of the same Femora (Figure 2).

## 3. Results

**Figure 3:**
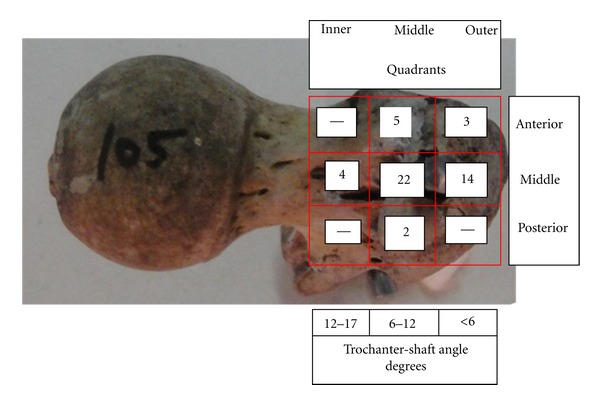
Showing distribution of exit of reamur through proximal and superior aspect in various quadrants in Saggital and Coronal plane.

In the anthropometric study, trochanter shaft angle 1 (TSA) measured maximum as 17 degrees and minimum of 5 degrees with average of 10.2 degrees. Radiological study it range from 4 to 14 degree with average 11.4 degrees. Overall in 27 cases (54%), exit points of reamur exit in the middle quadrant in sagittal and coronal plane, which corresponds to the trochanter-shaft angle range between 6–12 degrees both radiological and anthropometry, followed by outer-middle quadrant in 12 (24%), which corresponds to T-S angle <6 degree and inner middle in 3 (6%), which corresponds to T-S angle 12–17 degrees. anterior middle quadrant in five, anterior outer quadrant in three and posterior middle quadrant in two femora (Figure 3 distribution of exit of remur in coronal and Sagittal plane).

## 4. Discussion and Conclusion

 Conventional intramedullary nails, being flexible enough, provide the variation in entry point selection, hence Piriformis fossa, Greater trochanter, or any nearby point can be used without much inconvenience and complication. The uniplaner or biplaner rigid interlocking nails are less forgiving in selection of entry point and technically more demanding. Various complications [7–9] like iatrogenic fracture of neck, commnuition of the proximal part of femur, osteonecrosis of the femoral head, heterotrophic ossification around entry point and residual symptoms such as thigh and hip pains, limping, limited walking distance, and abductor weakness have been reported.

 The Piriformis fossa entry site [10–12] has been advocated as standard portal, but there are studies which have shown various difficulties and complications such as, difficulty in identifying and reaching to the piriformis fossa and malreduction, avascular necrosis of femoral head following damage to branches of medial circumflex femoral artery, injury to pelvi-femoral muscles (gluteus medius, gluteus minimums) and injury to branches of inferior division of superior gluteal nerve. Therefore, various other entry points like tip of the greater trochanter, lateral to tip of greater trochanter, and medial to greater trochanter have also been described, but their anatomical relationship with the geometry of the femur bone is not very clear. Therefore, we have conducted this anthropometric and radiological study on 50 dry cadaveric femora, to find out the anatomical basis of selection of entry point for uniplaner rigid antegrade interlocking nail. An anatomical point for the safe nail insertion of uniplaner rigid interlocking nail should lie in the line of medullary canal of femur, which proximally on trochanter area can be denoted with the help of trochanter shaft angle. We have not found any description of this angle in the literature. We have named this Trochanter-Shaft angle (TSA) as R-angle.

 Trochanter-shaft angle (TSA) measured on dry femora and Computerized digital radiograms of the dry cadaveric femora after nullifying the angle of torsion. Retrograde inserted the 7 mm. reamer through centre of intercondylar reamed to reach proximally; its exit point was noted in coronal and Saggittal plane by dividing the proximal superior femur (trochanter and adjacent area) in 9 quadrants area. 7 mm reamar was selected in the study because it is flexible enough to adapt in the natural curvature of the femur without making an inadvertent piercing pathway.

In anthropometric study, trochanter shaft angle (TSA) measured maximum as 17 degrees and minimum of 5 degrees with average of 10.2 degrees. In radiological study, it range from 4 to 14 degrees with average 11.4 degrees. The level of base of lesser trochanter marks the transition zone between cancellous to cortical bones that is, trochanter area and the shaft. It also serves as the level of determining the bent in case of biplaner nail. Distance from tip of greater trochanter to the base of lesser trochanter, in morphological study, is average 6.77 cms, and in radiological study, average distance is 6.73 cms, which is fairly comparable. Distance between the superior most point on the Greater trochanter and the Mid line of Shaft. This distance correlates with the area available for the proximal femoral nail diameter with safe nail insertion without fracture or damage to medial capsule and important vascular supply of femoral head. In anthropometric 1 study, it measured maximum 2.3 cm to 1 centimeter and in radiological measurement ranges from 2 to 0.95 cms. The degrees of angulation in the biplaner nail should be the half of the Trochanter-shaft angle, that is, R-Angle (as during reaming due to circumferential cut, entry point increases half of the angle on either side).

Grechenig et al. [10] studied the anatomy of the greater Trochanter and clinical importance in intramedullary femoral nailing in cadaver specimens and reported that trochanter may cover the actual entry site resulting in a much more medial entry site and recommended resection of parts of the Trochanter and preoperative CT scan, but this facility may not be available in all cases hence application of trochanter shaft angle is useful during surgery under image intensifier guidance. Ricci et al. [3], In their study, of trochanteric versus piriformis entry portal for the treatment of femoral shaft fractures, shows that piriformis fossa entry point has longer operative and fluoroscopic time. 

Ostrum et al. [4] conducted the analysis of the eccentric starting point for trochanteric intramedullary femoral nailing and found that the starting point seems to be the single most important factor, his study suggested that the coronal starting point be located at the junction of the anterior one-third and posterior two thirds of the trochanter; this correlates with our study in which most of the exit point of reamer emerges through middle quadrant 1 in coronal and Sagittal plane. A clinical study by Ostrum et al. [4] and cadaveric study by Grechenig et al. [10] and Ziylan and Murshid [13], reported that anatomy of the greater femoral trochanter is variable and site its clinical importance but how it is made to the clinical application was not clear. In our study, we tried to develop the correlation of variable anatomy of proximal femur (Trochanter) and its simple useful actual clinical application by measuring the trochanter-shaft angle (TSA) and its significance in deciding selection of entry point. Perez et al. [14] studied the Gluteus medius tendon injury in medial Trochanter entry Point.

The most important in selection of entry point is the alignment of the medullary canal of femur and its relationship with the corresponding point over proximal superior femur. We confirmed the alignment by retrograde insertion of reamer and notifying the exit of it through the proximal end of femur and further confirmed it by radiological analysis. While deciding the exact anatomical entry point, assessment of R-angle can serve as an important landmark for proper antegrade femoral nail insertion.

Georgiadis et al. [15] and Gausepohl et al. [11] in their study studied the entry point. In 88% of the specimen, the ideal entry point for a straight nail was found constantly at the medial border of the greater trochanter overlaying the tendinous insertion of the piriformis muscle. This point correlates with the middle quadrant in coronal and sagittal plane in our study. Dora et al. [16] in a cadaver study recorded the soft tissue damage in antegrade femoral nailing at entry point stated entry point selection should be ease of nail insertion and must be weighed against the resulting soft tissue damage. He ranked the entry site as the nail starting point at the piriformis fossa causing most damage to the external rotators also injuring branches of the medial femoral circumflex artery, the worst entry portal. Entry portals anterior to the longitudinal axis of the femoral neck and lateral, at the greater trochanter, cause far less damage to the pelvi trochanteric musculature and are safe as far as the ramus profundus of the medial femoral circumflex artery and its branches. In our study, we actually found that no reamer exit through the so-called piriformis fossa and exit site mostly correlates lateral and anterior to it in middle quadrant mostly.

Anatomical and radiological evaluation reveals that the entry point for antegrade femoral nail insertion is highly variable, and it cannot be defined in terms of piriformis fossa or greater trochanter. The entry point precisely correlates with the proximal femoral anatomy, the type of nail used (uniplaner or biplaner), anatomy of the trochanter itself, trochanter-shaft angle (R-Angle), medullary canal, and diameter of nail. If trochanter-shaft angle (R-Angle) is in between 0 degree to 6 degrees, the point of entry corresponds to the trochanteric region increase in Trochanter-shaft angle (6–12 degrees) corresponds to medial border of trochanter and further increase (12–17 degrees) shift the entry site more medial to greater trochanter which may risks the medial vascular structure and may need to choose biplaner nail for safety.

## Figures and Tables

**Table 1:** Data of morphological and radiological study.

				Distance between tip of			Distance from tip of greater		
	Trochanter-Shaft Angle degrees			Greater trochanter and base			trochanter to the mid-line of		
				of lesser trochanter cms.			shaft cms.		
	Max.	Min.	Avg.	Max.	Min.	Avg.	Max.	Min.	Avg.
Morphological Study	17	5	10.2	7.90	6.00	6.77	2.3	1.0	1.66
Radiological Study	14	4	11.4	8.09	1.64	6.73	2.0	0.95	1.30
